# Sexually transmitted infections in women in a rural hospital in Sierra Leone: a retrospective database study

**DOI:** 10.1016/j.ijregi.2025.100652

**Published:** 2025-04-17

**Authors:** Emmanuel Marx Kanu, Henning Rottmann, Ioana D. Olaru, Tom Theiler, Islam M. Kargbo, Hanna M. Mathéron, Laura C. Kalkman, Martin P. Grobusch, Frieder Schaumburg

**Affiliations:** 1 Masanga Medical Research Unit, Masanga Teaching Hospital, Tonkolili District, Sierra Leone; 2 Centre of Tropical Medicine and Travel Medicine, Department of Infectious Diseases, Faculty of Medicine, Amsterdam University Medical Centers, Location AMC, University of Amsterdam, Amsterdam, The Netherlands; 3 Institute of Medical Microbiology, University Hospital Münster, Münster, Germany; 4 Institute of Tropical Medicine & Deutsches Zentrum für Infektionsforschung, University of Tübingen, Tübingen, Germany; 5 Centre de Recherches Médicales, (CERMEL), Lambaréné, Gabon

**Keywords:** LAMP assay, Sexually transmitted diseases, Chlamydia trachomatis, Neisseria gonorrhoeae, Treponema pallidum, Sierra Leone

## Abstract

**Objectives:**

This study aimed to determine the frequency of sexually transmitted infections among women of reproductive age in a rural hospital in Sierra Leone.

**Methods:**

This retrospective cross-sectional database study included vaginal and endocervical swabs from 104 women of childbearing age (March 2023-March 2024) in Masanga Teaching Hospital, rural Sierra Leone. Samples were screened by loop-mediated isothermal amplification (eazyplex STD complete) for *Chlamydia trachomatis, Neisseria gonorrhoeae, Ureaplasma urealyticum, Mycoplasma hominis, Mycoplasma genitalium*, and *Treponema pallidum.* Demographic and medical data were obtained from medical records.

**Results:**

The median age was 26 years (range: 21-33 years). HIV status was available for 84 women, of whom nine (11%) were HIV-positive. Of the 75 women with clinical information available, 73 (97%) were symptomatic. Vaginal discharge (n = 66 of 75, 88%) was the most frequently reported symptom. Tests were positive for at least one target organism in the test panel in 54 of 104 cases (52%). *M. hominis* was most often detected (n = 47 of 104, 45%*),* followed by *U. urealyticum* (n = 18 of 104, 17%) and *N. gonorrhoeae* (n = 3 of 104, 3%), whereas *C. trachomatis* and *T. pallidum* were positive in one (1%) sample each. *M. genitalium* was not detected.

**Conclusions:**

The detection of obligate sexually transmitted infections pathogens (i.e. *C. trachomatis, N. gonorrhoeae, T. pallidum*; n = 5 of 104, 5%) was rare in our study population and setting.

## Highlights

• Sexually transmitted infections are still a major public health concern.
• A total of 5% of our study population tested positive for an obligate sexually transmitted infection pathogen.
• A total of 3% of our study population tested positive for *Neisseria gonorrhoea.*
• Loop-mediated isothermal amplification was the method of diagnosis.
• A total of 11% of our study population was positive for HIV.

## Introduction

Sexually transmitted infections (STIs) constitute a major public health problem. It is estimated that one million individuals per day acquire an STI caused by *Chlamydia trachomatis, Neisseria gonorrhoea, Treponema pallidum,* and *Trichomonas* sp. [1]. These infections are common in adolescents and adults and increase the risk for transmission of HIV [2]. Furthermore, STIs can cause a wide range of secondary complications (e.g. pelvic inflammatory disease, subfertility, obstetric, and neonatal complications) [3].

Worldwide, STIs are known to be endemic, particularly, in sub-Saharan Africa (SSA), where the number of incident cases was estimated at 86 million cases in 2016, second only to the Western Pacific region with 108 million cases [3]. Its prevalence is also known to vary widely within and between communities in SSA, ranging from 32.7% in Mali to 0.3% in Ghana [4]. These variations have been attributed to a multitude of socio-cultural, behavioral, and host-pathogen interaction factors, as well as specificities of country-level interventions [4].

A few studies were conducted in Sierra Leone, indicating a higher prevalence of symptoms suggestive of STIs among individuals with multiple sexual partners [5,6]. Despite these reports, the epidemiology of STIs in Sierra Leone remains by-and-large to be elucidated further. This study aims to address this knowledge gap by examining the prevalence of STIs among women of reproductive age at a district referral hospital in rural Sierra Leone.

## Material and methods

This retrospective cross-sectional database study was conducted from March 2023 to March 2024 at Masanga Teaching Hospital, Tonkolili District, Sierra Leone, a 120-bed secondary level of care facility.

The study included female patients of childbearing age who presented with clinical signs and symptoms suggestive of STI. Vaginal or endocervical swabs were collected and examined for the presence of STI. Testing and request decisions were made at the discretion of the treating physician. Age, sex, in- or out-patient status, self-reported HIV status, and type of signs and symptoms (i.e. vaginal discharge, dysuria, genital ulcers, lower abdominal pain, dyspareunia, and vaginal itching) were documented on a standardized laboratory request form for each specimen. Non-vaginal/endocervical swabs, duplicate, or follow-up samples were excluded.

Vaginal or endocervical swabs (Sigma Transwab, liquid Amies medium, MWE, Corsham, Wiltshire, UK) were tested within 30 minutes after sampling by loop-mediated isothermal amplification (LAMP) (eazyplex STD complete, AmplexDiagnostics, Gahrs-Bahnhof, Germany) on a Genie II (AmplexDiagnostics), following the manufacturer’s instruction. This test can detect *C. trachomatis, N. gonorrhoeae, Ureaplasma urealyticum, Mycoplasma hominis, Mycoplasma genitalium*, and *T. pallidum* [7].

The treating physician prescribed targeted antibiotic therapy to patients whose samples had tested positive for an obligate STI pathogen, in accordance with the local antimicrobial guideline. Patients with genital ulcers for whom a clear cause could not be identified using the LAMP assay were given syndromic management, whereas those who tested positive for commensal were not prescribed antibiotics. Proportions were compared using the chi-square or Fisher’s exact test, as deemed appropriate, using R, version 4.4.1 [8].

## Results

Samples from 104 women of childbearing age with a median age of 26 years (range: 21-33 years) were included in this analysis (Table 1). The majority of our study population were in-patients (n = 62 of 104; 60%) who were admitted into the maternity ward. Self-reported HIV status was available for 84 of 104 (81%) women, of whom nine of 84 (11%) were HIV-positive. Of the women with available clinical information, 73 of 75 (97%) were symptomatic. Symptoms reported were vaginal discharge (n = 66 of 75; 88%), dysuria (n = 22 of 75; 29%), genital ulcers (n = 20 of 75; 27%), lower abdominal pain (n = 16 of 75; 21%), dyspareunia (n = 11 of 75; 15%), and vaginal itching (n = 3 of 75; 4%) (Table 1).

**Table 1:** Demographic and test characteristics and signs and symptoms of patients.

	Variables	STIs
**Demographic characteristics**	Included patients (n)	104
	Median age (years [interquartile range])	26 (21-33)
	Proportion of out-patients (% [n])	40% (42)
	Proportion of self-reported HIV infection (% [n])	11%^a^ (9)
	Proportion of positive test (%[n]) Proportion of women with two or more target pathogens detected (% [n])	52% (54) 14% (15)
	Proportion of true STI pathogens detected (%[n])	5% (5)
**Symptoms of STIs**	Number of included patients with clinical information	75
	Vaginal discharge (% [n])	88% (66)
	Dysuria (% [n])	29% (22)
	Genital ulcer (% [n])	27% (20)
	Lower abdominal pain (% [n])	21% (16)
	Dyspareunia (% [n])	15% (11)
	Vaginal itching (% [n])	4% (3)

STI, sexually transmitted infections.

a A total of 84 of 104 patients in the study population had a documented HIV status.

Tests were positive for at least one target organism in the test panel in 54 of 104 (52%) of cases; 15 of 104 (14%) of women had two or more target pathogens detected (Table 1). Five women had an obligate STI pathogen detected (n = 5 of 104; 5%) (Table 2).

**Table 2:** Organisms detected in the study population (n = 104).

Organism	Proportion
a*Mycoplasma hominis* [% (n)]	45 (47)
a*Ureaplasma urealyticum* [% (n)]	17 (18)
b*Neisseria gonorrhoea* [% (n)]	3 (3)
b*Chlamydia trachomatis* [% (n)]	1 (1)
b*Treponema pallidum* [% (n)]	1 (1)
b*Mycoplasma genitalium* [% (n)]	0 (0)

a Organisms considered as commensal of the female genital tract.

b Organisms considered as obligate pathogens of the female genital tract.

## Discussion and conclusion

The prevalence of STIs caused by *C. trachomatis, N. gonorrhoeae,* and *T. pallidum* was low in our study (5%), with vaginal discharge being the most reported symptom in 88% of cases. A few studies conducted in Sierra Leone have indicated a higher prevalence of STIs at 21.6% and 32%, respectively. It is, however, noteworthy that the findings of these studies are based on self-report [5,6]. A systematic review has also demonstrated an increased prevalence of *M. genitalium* (18.0%), *C. trachomatis* (14.7%), and *N. gonorrhoeae* (4.4%) in Southern Africa, although studies using molecular diagnosis were scarce [9]. To the best of our knowledge, our study is the only published report on LAMP assay of STIs in Sierra Leone. Although *M. hominis* and *U. urealyticum* were the most common species, they are commensals of the genital tract and infrequently reported to be causing severe infections in neonates [10]. It remains controversial to include these organisms within testing panels because they are usually not considered pathogenic, thus rendering interpretation of test results difficult [11]. The prevalence of *N. gonorrhoeae* of 3% in our study population is consistent with meta-analytic data from SSA, where a prevalence of 3.3% has been reported in women of reproductive age [12]. Assuming a high number of asymptomatic carriers with limited access to health systems with low testing capacity in rural Africa, this underscores the need for standardized and expanded testing strategies. In addition, the relatively high HIV prevalence (11%) reported here contrasts with the national prevalence of 1.7% [13]. We attributed this to the enhanced STI testing capacity at Masanga Teaching Hospital, which facilitates more HIV tests because clinicians would frequently request to rule out co-infections.

The comparatively low prevalence of STIs in symptomatic women in our study population may be attributable to several factors. First, the study did not include the testing for other pathogens such as *Trichomonas* sp., human papilloma virus, or herpes simplex virus. Second, the symptoms may have been indicative of other conditions, such as urinary tract infections, bacterial vaginosis, or cervical cancer. Third, vaginal discharge can be approached from the perspective of a physiologic occurrence. The color, odor, consistency, quantity, and duration of vaginal discharge, which might have enabled more precise distinctions between normal and abnormal vaginal discharge, were not included in the data set.

Our study has limitations. The retrospective design of this study renders it susceptible to missing data and incomplete documentation, as well as impeding further statistical analysis. The proportion of specimen (vaginal vs endocervical swab) could not be ascertained due to ambiguity in the laboratory forms submitted by the treating physicians. Although vaginal swabs appear to be superior to cervical swabs for the detection of STI pathogens by nucleic acid amplification test (e.g. LAMP), the difference between the two samples is low. We, therefore, rate the risk of a sample bias as low [14]. Previous antibiotic use is difficult to accurately assess and was, therefore, not recorded. Furthermore, the testing for other STI beyond those encompassed within the panel (e.g. alternative diagnoses of genital ulcer such as herpes simplex virus 1 or 2, lymphogranuloma venereum, or chancroid) and the absence of concurrent urine cultures were notable shortcomings. Moreover, we might have underreported positive cases because the reported sensitivity of the applied LAMP assay can be low (70-88%) [7].

In conclusion, the proportion of obligate STI is low. Although this may offer reassurance to a rural population, it does, nevertheless, highlight the necessity for the conduct of a comprehensive prospective research to determine which population in our setting is at risk of contracting an STI and its complications.

## Declarations of competing interest

The authors have no competing interest to declare.
